# Biopolymer/Suture Polymer Interaction: Is It a Key of Bioprosthetic Calcification?

**DOI:** 10.3390/polym17111576

**Published:** 2025-06-05

**Authors:** Irina Yu. Zhuravleva, Anna A. Dokuchaeva, Andrey A. Vaver, Ludmila V. Kreiker, Elena V. Kuznetsova, Rostislav I. Grek

**Affiliations:** 1 Institute of Experimental Biology and Medicine, E. Meshalkin National Medical Research Center of the Ministry of Health of the Russian Federation (E. Meshalkin NMRC), 15, Rechkunovskaya str., Novosibirsk 630055, Russia; a_dokuchaeva@meshalkin.ru (A.A.D.); vaver_a@meshalkin.ru (A.A.V.); kreyker_l@meshalkin.ru (L.V.K.); ev_kuznetsova@meshalkin.ru (E.V.K.); 2 Icon Lab Gmbh Ltd., 1 Barrikad St., Nizhny Novgorod 603003, Russia; grek@iconlab.ru

**Keywords:** bioprosthetic heart valves, collagen, REPEREN^®^, suture material, calcification

## Abstract

The aim of this study was to evaluate the effect of suture material made of polyester (PET), polypropylene (PP), and polytetrafluoroethylene (PTFE) on the calcification of a bovine pericardium (BP) consisting of collagen biopolymer preserved with an epoxy compound. Non-porous film made of the synthetic reinforced polymer REPEREN^®^ was chosen as a control material. Samples of the material (sutured or non-sutured with each of the three types of surgical sutures) were implanted subcutaneously in 45 young rats for 30, 60, and 90 days. The calcium content of the explants was quantified using atomic absorption spectrometry, a histological examination was performed using hematoxylin and eosin and von Kossa staining, and the structure of the calcium phosphate deposits was studied using scanning electron microscopy (SEM) and energy dispersive spectrometry (EDS) with color field mapping. The results demonstrated the absence of calcification in the non-sutured BP and in all the REPEREN^®^ groups. In the sutured BP samples, a dynamic increase in the Ca content and the Ca/P ratio to 1.67–1.7 (crystalline hydroxyapatite) was observed by the 90th day. The minimum Ca content among the sutured BP groups was detected in samples where the PET thread was used. The cellular reaction to BP was significantly more pronounced than the reaction to REPEREN^®^ throughout the entire observation period; collagen homogenization was noted near the sutures. It can be concluded that all the studied suture materials provoke BP calcification. PET has the minimal negative effect.

## 1. Introduction

Bioprosthetic heart valves have taken place in cardiac surgery since the early 1970s, after A. Carpentier proposed preserving xenogeneic material with glutaraldehyde (GA). However, the first long-term and mid-term results of operations showed that bioprostheses have a significant drawback: GA-treated xenotissue has high calcium-binding activity, and the calcium phosphate deposits formed in it are transformed over time into massive hydroxyapatite deposits, which leads to bioprosthesis dysfunction [1]. The only solution to this situation is re-operation with replacement of the failed bioprosthesis. In addition, in the 1980s, cardiac surgeons noted that calcification of bioprostheses accelerates in young patients, pregnant women, and individuals with concomitant diseases accompanied by impaired calcium metabolism (chronic renal failure, hyperparathyroidism, etc.) [1,2,3]. These observations have led to very limited indications for the use of bioprostheses. Both European and American guidelines recommend implanting bioprostheses in patients over 65 years of age [4], and for younger patients the decision is made individually in each case based on a comparison of the various risks.

Despite the fact that the connection between GA cross-linking and bioprosthetic calcification has been repeatedly proven and is currently beyond doubt [1,5,6], all commercial valves are still preserved with GA. Historically, many methods have been proposed to protect bioprosthetic tissues from calcification, including GA-free crosslinking [7,8,9]. However, only two techniques have reached clinical application: Lynx and ThermaFix, based on primary crosslinking with GA [10,11].

In this regard, Russian surgeons have unique experience with epoxy-treated bioprostheses. Ethylene glycol diglycidyl ether (diepoxyde, DE) has been used by NeoCor company for biologic material cross-linking since 1995 [12]. The scientific literature from the early 1990s and our own experimental studies proved that the calcium-binding capacity of epoxy-treated bioprosthetic materials is minimal (porcine aortic valve leaflets) or absent (bovine pericardium). Furthermore, epoxy-treated xenogeneic materials have been shown to be more bio- and cyto-compatible than GA counterparts [13,14,15,16,17,18]. However, subsequent clinical results have shown that epoxy treatment did not completely prevent calcification, although these bioprostheses were more resistant to this complication [19,20,21].

**Figure 1:**
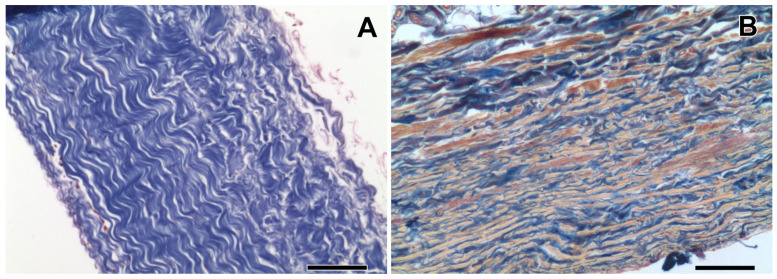
Tissue composition and structure of bovine pericardium (**A**) and jugular vein wall (**B**). Mallory trichrome staining: collagen is blue, elastin is yellow, and smooth muscle and other cells are red. Scale bar: 50 μm.

As for the elastin-containing materials (porcine aortic and bovine jugular vein walls), the clinical results correspond to the experimental ones [22]. Previously, we repeatedly observed the voluminous calcification of these materials treated with DE during subcutaneous implantation in rats, although at a lower calcium content than in GA counterparts [18,23]. This is explained by the fact that the elastin contained in these materials (aortic wall, bovine vein, valve cusp), on the one hand, does not interact with cross-linking agents such as GA and DE, remaining in its natural state [24], and on the other hand, it contains a large number Ca-binding sites [25]. However, bovine and porcine pericardia contain virtually no elastin, their main biopolymer is collagen (Figure 1). In the rat model, the epoxy-treated pericardium never was calcified, unlike the GA-treated pericardium [17,18,23]. Thus, it can be concluded that the natural biopolymer collagen cross-linked with DE does not have calcium-binding properties. Of course, this is limited to the collagen retaining its normal structure; when its fibrillar structures are damaged or homogenized, collagen derivatives behave unpredictably due to the appearance of new free reactive groups as a result of biopolymer destruction.

In this regard, it is of great interest to compare the experimental results with clinical experience with the pericardial right-sided valved conduits [26,27,28,29]. In this intracardiac position, calcification factors such as high hemodynamic load (where the valve closing pressure does not exceed 15 mm Hg) are minimized, which excludes primary fatigue damage to collagen with its subsequent destruction and the appearance of calcium-binding sites in collagen derivatives. The follow-up of patients operated in our clinic showed that calcification of the pericardial conduits along the suture line is a fairly common phenomenon (Figure S1). At the same time, the valve apparatus and the rest of the conduit wall are normal. Typically, hemodynamics is not affected and re-do surgery is not required. These results are typical for both the pediatric group (Figure S1A,B) and young adults (Figure S1C,D).

This suggests that in DE pericardial conduits, the implant calcification may be associated with the suture material. It remains to be seen whether this is due to the direct toxic effect of the suture material, a cellular attack on it, features of the immune response, or a combination of factors. Will we obtain evidence of the negative effect of the suture material in a model experiment on rats? How can the suture material affect other types of polymers (e.g., synthetic polymer films)? These questions have not been studied previously but should be studied.

The aim of this study was to evaluate the effect of the main suture materials used in cardiac surgery (polyester (PET), polypropylene (PP), and polytetrafluorethylene (PTFE)) on the calcification of a bovine pericardium consisting of collagen biopolymer preserved with an epoxy compound. A film made of the non-porous synthetic polymer REPEREN^®^ was used as a control material.

## 2. Materials and Methods

### 2.1. Materials

For a comparative study of the calcification dynamics, samples made of biological material (biopolymer) and the synthetic non-porous polymer REPEREN^®^ (IconLab Gmbh, Nizhny Novgorod, Russia) were used. As a biopolymer, we the chose bovine pericardium (BP), which consists almost entirely of collagen [17].

Ethylene glycol diglycidyl ether (98% purity, MW 218 Da) purchased from the N. Vorozhtsov Novosibirsk Institute of Organic Chemistry, SB RAS (Novosibirsk, Russia) was used as a cross-linking agent. The fresh bovine pericardium was obtained from healthy animals immediately after slaughter, rinsed several times with 0.9% NaCl, and then cross-linked at room temperature using 5% DE (0.1 M phosphate buffer, pH 7.4 for 14 days, with one solution change on the 3rd day). The preserved BP was stored in a complex solution containing 1% antimicrobial agent (1,2-octanediol, phenoxyethanol, sorbic acid) and 20% ethanol [30].

REPEREN^®^ is a spatially cross-linked polymer synthesized from methacrylic oligomers reinforced with ultra-thin (50 micron) polyamide fibers made in a sandwich style (polyamide fibers inside and REPEREN^®^ on both sides). The film has one ultra-smooth side and the other side that is conditionally rough. We chose the 160 μm thick films to make the valves. Samples 85 × 85 mm were sterilized with ethylene oxide in “cold” mode (37 °C). The mechanical properties, hemo- and cytocompatibility, hydrophilicity, and calcium-binding capacity of REPEREN^®^ were studied previously by us [31].

Round 6 mm samples were cut from BP films with a thickness of 0.3–0.4 mm and REPEREN^®^ films with a thickness of 0.16 mm using a laser cutting machine (Figure S2) “MELAS-Cardio” (Institute of Laser Physics of the Siberian Branch of the Russian Academy of Sciences, Novosibirsk, Russia).

**Figure 2:**
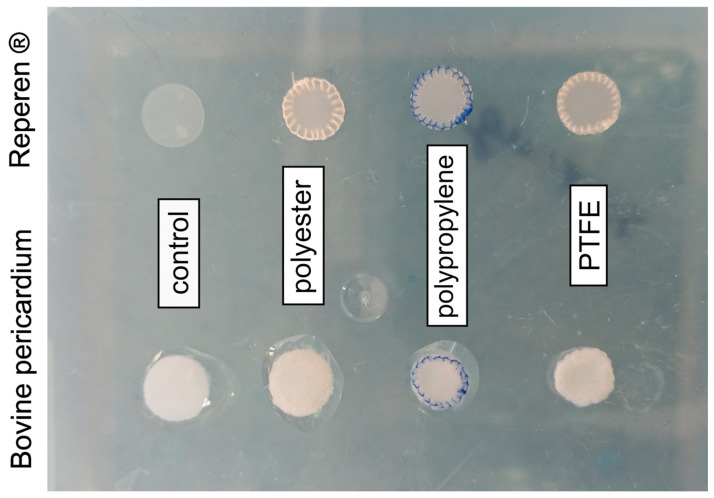
Sample types tested in this study.

Control samples of BP and REPEREN^®^ were not sutured; the other samples were sutured with continuous lock-stitch sutures (Figure 2) using white braided polyester 6-0, blue monofilament polypropylene 6-0, and white monofilament PTFE 6-0 (“MZKRS Suture Materials, Ltd.”, Moskow, Russia).

### 2.2. Subcutaneous Implantation in Rats

All the experimental procedures were performed in accordance with the EU Directive 2010/63/EU for animal experiments and approved by the Ethics Committee of the E. Meshalkin National Medical Research Center.

Four-week-old male Wistar rats (40–50 g, *n* = 45) were anesthetized with 50 mg/kg Telazol (Zoetis Manufacturing & Research Spain, S.L, Gerona, Spain). Each animal was implanted with 8 samples (4 samples of each material). Eight incisions were made on the dorsal surface to prepare subdermal pouches. Each pouch was filled with one REPEREN^®^ or BP sample and closed with one stitch. Fifteen samples of each biomaterial type were explanted on days 30, 60, and 90 and rinsed with 0.9% NaCl. One half of each sample type was used for histological and SEM/EDS studies, and another half underwent calcium content analysis.

### 2.3. Histological Studies

All the explanted samples for conventional histological studies were explanted with surrounding tissue capsules, fixed in 10% neutral buffered formalin, embedded in paraffin, and then cut into 6 μm thick slides. All the samples were stained with H&E and Von Kossa methods.

### 2.4. Scanning Electron Microscopy (SEM) and Energy Dispersive Spectrometry (EDS) Analysis

Each explanted sample was cleared of surrounding tissue, straightened, fixed, and dried at room temperature under sterile conditions. Before testing, the samples were coated with a 25–30 nm thick conductive carbon layer using a GVC-3000 Thermal Evaporation Carbon Plating Instrument (KYKY Technology Co., Ltd., Beijing, China).

**Figure 3:**
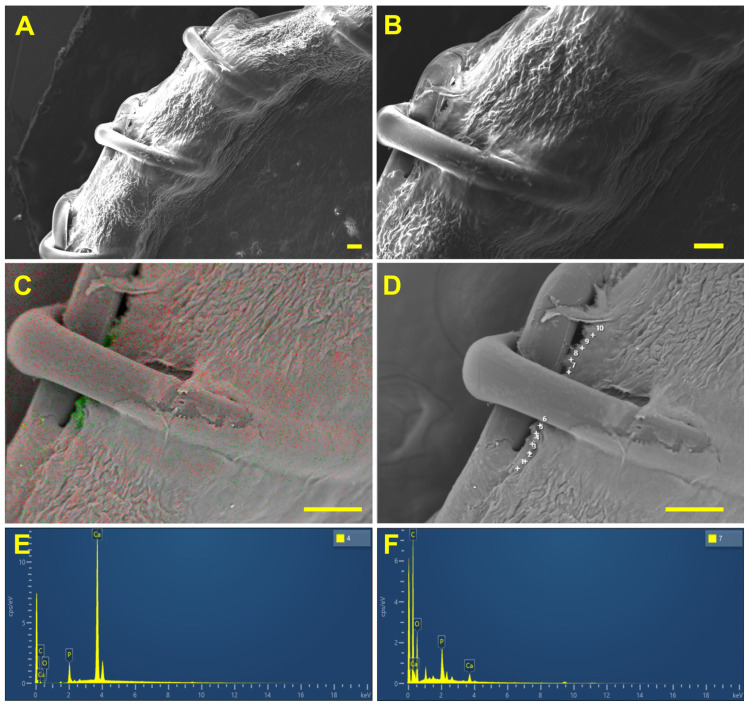
The example of SEM and EDS sequences. Overview images of the field at 50× (**A**) and 100× (**B**). Ca and P mapping (**C**); EDS analysis at marked points (**D**). Bottom images are the obtained spectra at points 4 (**E**) and 7 (**F**). The sample is “BP + polypropylene sutures” implanted for 30 days. Scale bars: 100 μm.

SEM and EDS analysis and elemental mapping of selected areas were carried out using a WIN SEM A6000LV scanning electron microscope (KYKY Technology Co., Ltd., Beijing, China) equipped with EDX system AzTec One (Oxford Instruments, Abingdon, UK) and AztecOne 6.0 SP2 software (Oxford Instruments, Abingdon, UK). At first, sample observation was performed using a secondary electron (SE) detector (Figure 3A). Ten observation fields for each sample were examined at 50×, 100× or 200× magnification. A field in the contact zone of the ligature and the BP or the polymer film was selected (Figure 3B). Then, using a back-scattering electron (BSE) detector at a high electron voltage of 20 keV and an electron beam setting of 120 μA, mapping for Ca and P was obtained (Figure 3C). The EDS analysis was performed in a spot mode (Figure 3D) at 15–40 points; atomic % was calculated automatically by AztecOne 6.0 SP2 software.

### 2.5. Calcium Content Analysis

The explanted samples were dried, weighed, and hydrolyzed in 14 M HNO_3_. Calcium quantification was conducted using a Thermo Solaar M6 atomic absorption spectrophotometer (Thermo Fisher Scientific, Waltham, MA, USA).

### 2.6. Statistical Analysis

Statistical analysis was performed using STATISTICA 10.0 software (StatSoft Inc., Tulsa, OK, USA). The Shapiro–Wilk test was applied to check the normality of distribution in each group. Since the distribution of quantitative characteristics in most groups was not normal, non-parametric statistics were used, and the data are reported as medians (Me) and interquartile ranges (25–75%) (IQRs). The Mann–Whitney (M–W) U-test was used to compare the two groups. The significance level was set to *p* < 0.05.

## 3. Results

### 3.1. Calcium Content in Biomaterials

**Figure 4:**
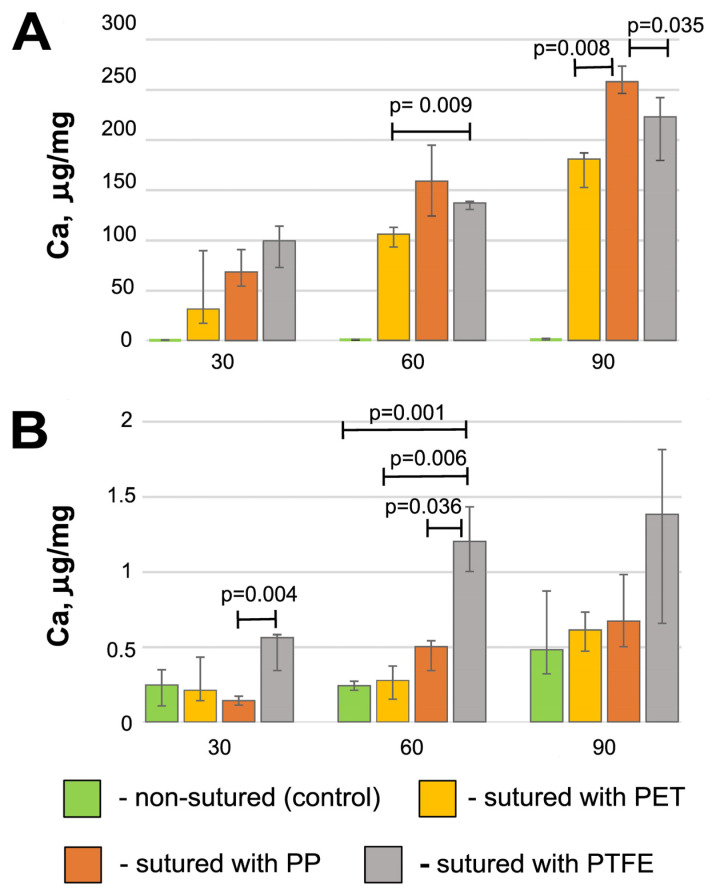
Calcium content in DE-treated bovine pericardium (**A**) and REPEREN^®^ (**B**).

The obtained results demonstrated the absence of significant calcium accumulation in the control (non-sutured) BP. Over a period of 90 days, the amount of this element was 0.36–1.89 μg/mg dry tissue. At the same time, the calcium concentration in the sutured samples progressively increased (Figure 4A). The maximum amount of calcium (257.5 (245.9; 273.0) μg/mg) accumulated by day 90 in the BP sutured with polypropylene. This is 30% more (*p* = 0.008) than in the samples sutured with polyester, and 19% more (*p* = 0.035) than in the samples sutured with PTFE. Despite these differences, it should be noted that in all the sutured samples the calcium level is “clinically significant” and approximately corresponds to the calcified valves removed from patients after 5–10 years of functioning [32].

Completely different data were obtained in all the REPEREN groups (Figure 4B). Over 90 days, the calcium concentration did not rise above 1 μg/mg, with the exception of the PTFE-sewn samples, where the calcium level increased to 1.5 μg/mg on the 60th and 90th days. However, if on the 60th day the differences between PTFE-REPEREN and the other film groups are significant (*p* < 0.003), by the 90th day no significant difference was found (*p* > 0.05), which is mainly due to the large individual variability of the Ca content, although the trend remains.

### 3.2. Histology Results

**Figure 5:**
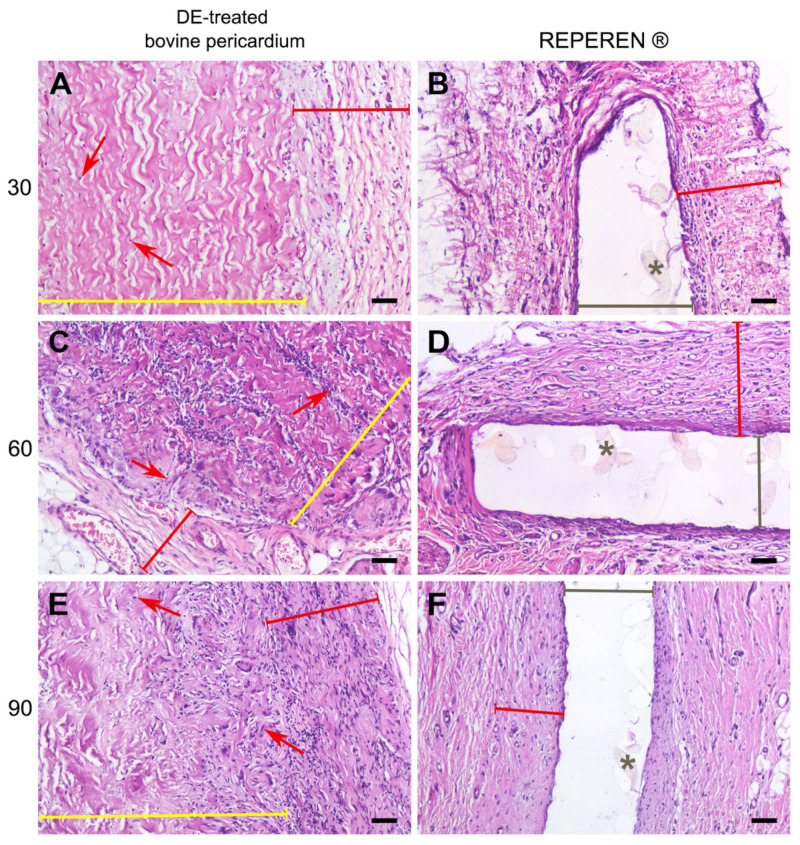
Cell response to control samples of BP (**A**,**C**,**E**) and REPEREN (**B**,**D**,**F**) implants. Yellow lines indicate DE-treated pericardium tissue, red lines indicate the surrounding fibrous capsule, gray lines indicate REPEREN film, and gray asterisks indicate polyamide fibers in the REPEREN film. Red arrows—recipient cells migrating between the collagen fibers of pericardial samples. Scale bars: 50 μm.

The histological examination showed that the DE-treated control pericardium induced a certain tissue reaction, but it was virtually absent in the REPEREN control samples (Figure 5). In the DE-treated BP group, cell migration occurred into the deep collagen layers by day 60 (Figure 5C). These were, in particular, cells of the lymphocyte family, as well as fibroblasts of varying maturity. The cell penetrating into the sample indicates the beginning of the implant reorganization and replacement; fibrous threads began to form between the collagen fibers, which by day 90 created a network intertwined with the sample material (Figure 5E). Since the pre-implanted BP did not contain any cells, it can be stated that these cells visualized in the layers of the implant migrated from the recipient’s tissues. In the 60-day BP group, single foreign body giant cells (FBGCs) appeared at the border of the fibrous capsule and the implant. Their number did not increase further, no implant rejection occurred, and the tissue reaction to this material led to its partial replacement by the recipient’s own tissues.

REPEREN appeared to be a histo-compatible, low-reactive material that is not subject to biodegradation or replacement by the recipient’s tissues. At all observation time points, no FBGCs or foci of pronounced lymphocytic infiltration were detected in these samples, and the implant was surrounded by a uniform, dense connective tissue capsule. Concerning the other time points, it is noteworthy that the only visualized changes in the REPEREN groups (30, 60, and 90 days) addressed the proliferation of the formed fibrous capsule. Visible signs of graft rejection, inflammation, or tissue mineralization were absent in all samples with or without suture material (Figures S3–S5).

**Figure 6:**
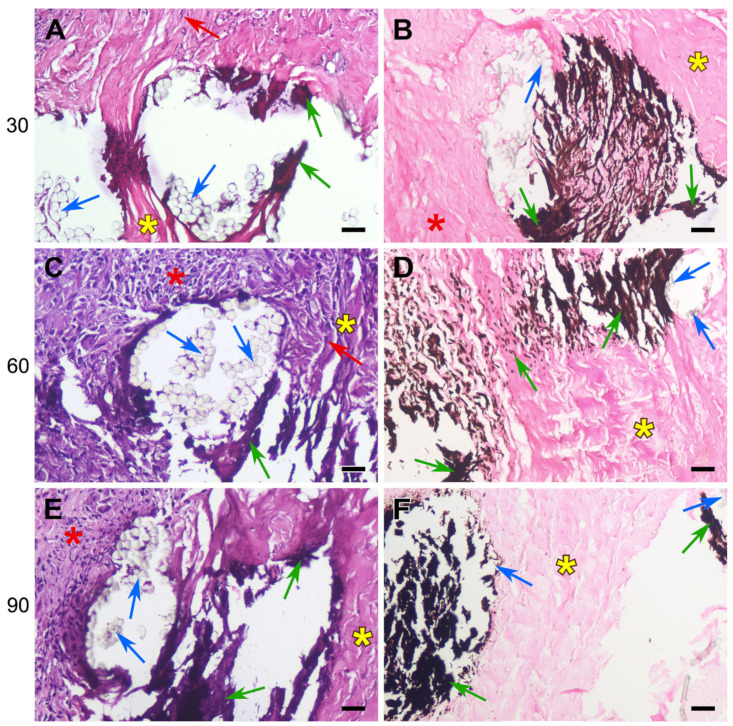
Calcification dynamics in the polyester sutured BP implants. H&E (**A**,**C**,**E**) and von Kossa (**B**,**D**,**F**) staining. Yellow asterisks indicate DE-treated pericardium tissue; red asterisks indicate the surrounding fibrous capsule. Red arrows indicate recipient cells migrating. Blue—suture material; green—tissue mineralization. Scale bars: 50 μm.

**Figure 7:**
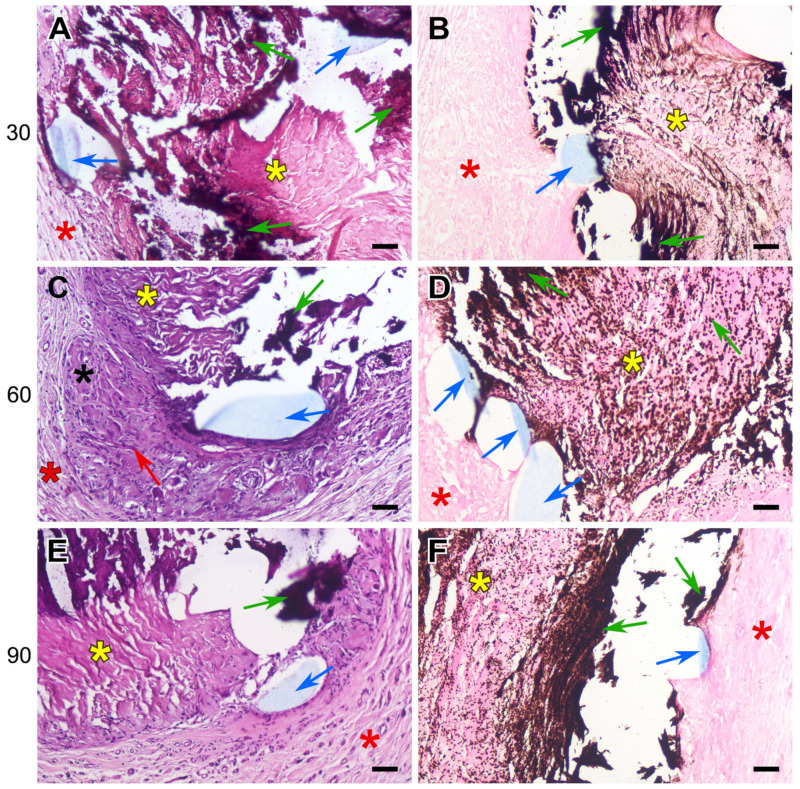
Calcification dynamics in the polypropylene sutured BP implants. H&E (**A**,**C**,**E**) and von Kossa (**B**,**D**,**F**) staining. Yellow asterisks indicate DE-treated pericardium tissue; red asterisks indicate the surrounding fibrous capsule; black asterisks indicate the newly formed fibrous tissue. Red arrows indicate recipient cells migrating. Blue —suture material; green—tissue mineralization. Scale bars: 50 μm.

**Figure 8:**
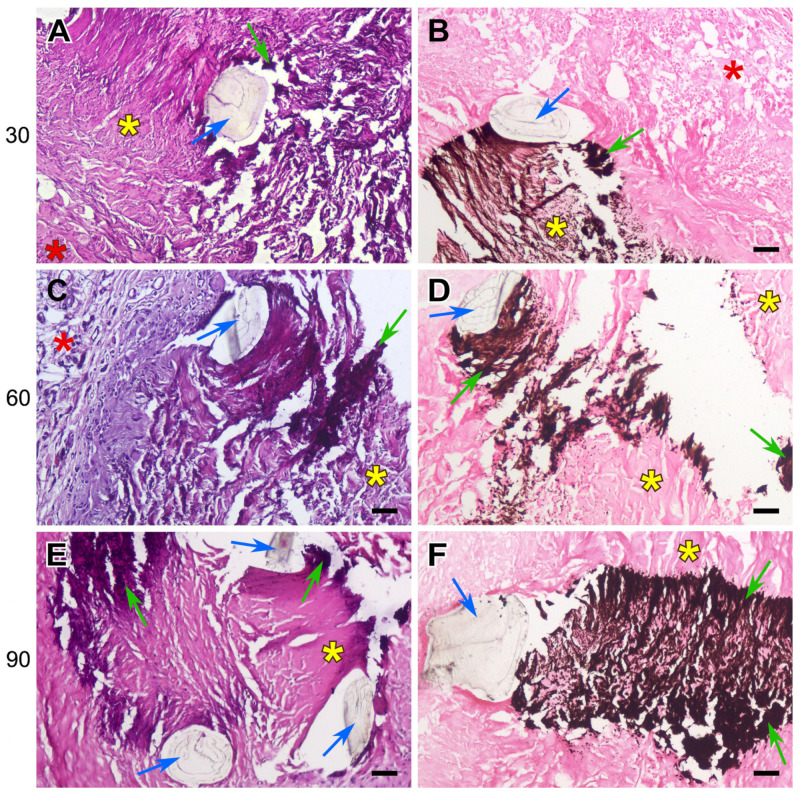
Calcification dynamics in the polytetrafluorethylene sutured BP implants. H&E (**A**,**C**,**E**) and von Kossa (**B**,**D**,**F**) staining. Yellow asterisks indicate DE-treated pericardium tissue; red asterisks indicate the surrounding fibrous capsule. Blue arrows indicate suture material. Green–tissue mineralization. Scale bars: 50 μm.

The sutured DE-treated BPs showed the same histological picture as that of the control samples of the biomaterial: moderate lymphocyte infiltration, the proliferation of fibrous strands in the implant layers, the migration of recipient cells, and the appearance of FBGCs. However, in addition to the general histological picture of biomaterial restructuring, signs of collagen homogenization and tissue mineralization appeared by the 30th day. Calcification areas were localized along the suture material, which was easily noticeable even at very low magnification. The pattern formed by mineral accumulations repeated the shape of the suture lines, which is well visualized with specific von Kossa staining (Figure 6, Figure 7 and Figure 8).

The calcification of collagen fibers in the DE-treated pericardium begins with individual scattered spots (mineralization points), which then merge, spreading along the homogenized collagen fibers, gradually covering the entire bundle, which is clearly visible when using specific dyes (Figure 6, Figure 7 and Figure 8).

On the 30th day after implantation, hard, fragile calcium phosphate conglomerates surrounding the surgical thread formed at the border of the implant and the recipient’s connective tissues. If the braided polyester was used, crystals of calcium salts could either encrust the fibers of the thread or locate between them, but no incrustation was seen with monofilament polypropylene or PTFE threads. The mineralization degree shown by the samples containing all types of suture was almost the same at the 30th day; but by 60 and 90 days, the most extensive deposits of calcium salts were found in the polypropylene-sewn samples. The areas of homogenized collagen are most susceptible to mineralization, which is clearly visible in Figure 6A, Figure 7E and Figure 8B,E.

### 3.3. SEM and EDS Results

The EDS analysis and mapping of the studied samples for Ca and P supplemented the histological results with data on the structural transformations of calcium phosphate deposits in the studied samples of DE-treated BP.

On the 30th day, the changes were similar regardless of the suture material. Calcification foci were localized directly in the implant material, near the ligature (Figure 3C). They had a “mosaic” structure; that is, in some points of the deposit Ca predominated (Figure 3, left spectrum), while in others P (Figure 3, right spectrum) predominated. Accordingly, the Ca/P ratios at different points varied in wide intervals (Table 1). When using PET, a minimum Ca/P ratio was noted, which indicates the predominance of phosphates in the deposit structure. When using PTFE, the median Ca/P ratio already at this time point reached 1.57 and approached that in hydroxyapatite (the classic Ca/P ratio in crystalline hydroxyapatite is 1.67), despite the wide variability of the structure at different points.

**Figure 9:**
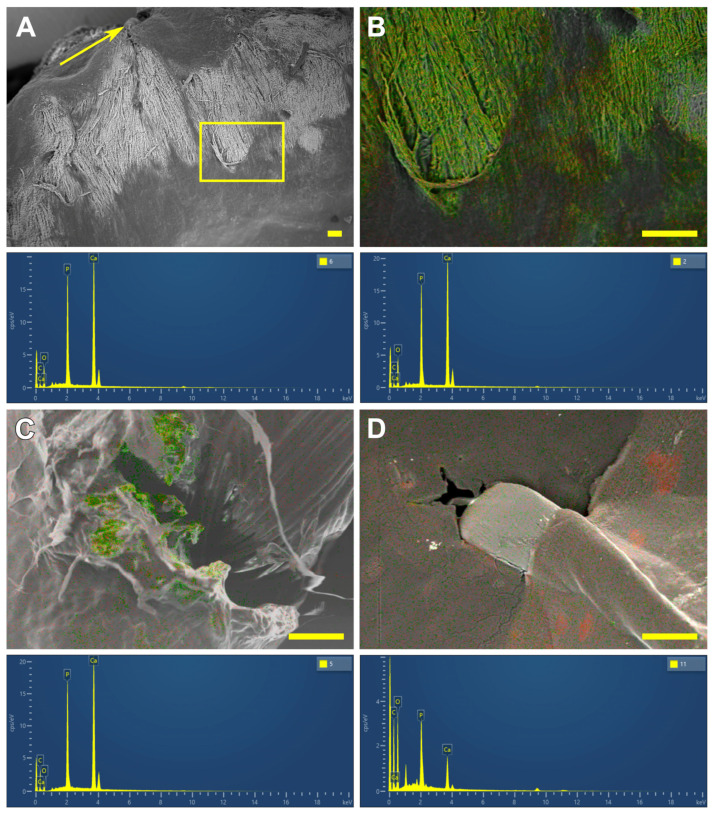
SEM image (BSE) of the BP sample sutured with PTFE after 60-day implantation (**A**). The arrow points to the suture; the field in a rectangle is mapped at (**B**). The spectra below these figures demonstrate the homogeneity of the calcium phosphate deposits (Ca/P ratios are 1.63 (left spectrum) and 1.71 (right spectrum). The EDS map of the 90-day BP implant sutured with PET; the spectrum below reflects the Ca/P ratio 1,67 (**C**). The EDS map of the 90-day REPEREN implant sutured with PTFE; the spectrum below reflects the Ca/P ratio 0.6 (**D**). Scale bars: 100 μm.

By the 60th day, a tendency towards the “maturation” of calcium phosphates was noted: regardless of the suture material used, the Ca/P ratio increased, tending towards the values of crystalline hydroxyapatite. In the PP and PET sutured samples, the structure of the deposits remained “mosaic”, which indicates the ongoing process of crystal formation. However, when using PTFE, the process of crystalline formation apparently ends by this time, since EDS mapping of the samples shows that collagen is completely mineralized in large areas, and the average Ca/P ratio is 1.7 (Figure 9A,B and the spectra below them).

A similar picture was observed by the 90th day in the samples sewn with PET (Table 1, Figure 9C, and the spectrum below it); however, calcium phosphate deposits captured smaller areas, at least on the surface of the samples. When using PP, the structure of deposits remained “mosaic” even on the 90th day, which indicates further potential for the formation of crystallization centers and the progression of calcium binding.

It should be emphasized that, although the REPEREN films themselves were not calcified throughout the entire period, the Ca-P deposits with a large predominance of P were found in some connective tissue capsules formed by the surrounding tissues of the recipient by the 90th day (Table 1, Figure 9D and spectrum, Figure S6).

## 4. Discussion

In this work, we have shown that the calcification of cardiovascular prostheses depends on both the biomaterial itself and the suture material used in their manufacturing. Histological results proved that the foreign body reaction (FBR) underlies the process of the mineralization of the DE-treated collagen biopolymer, and only if the implant contains suture material. The collagen implant treated with DE causes a typical FBR (Figure 5): a lymphocyte attack, the appearance of FBGCs, and fibroblast infiltration with the subsequent partial fibrosis of the implant [33]. However, calcium phosphate deposits, which are transformed into mature hydroxyapatite by the 90th day, appear only in samples containing suture material. The main feature of these samples compared to those that are not sutured is the partial lysis and homogenization of fairly large areas of collagen near the surgical thread with the subsequent mineralization of these areas (Figure 6, Figure 7 and Figure 8). At the same time, in the implanted REPEREN film, the severity of the FBR is minimal; the presence of suture material does not enhance it and does not promote mineralization (Figure 5 and Figures S3–S5).

We suppose that these differences are primarily related to the porosity of the implant material. Kyriakides TR et al. identified this property as the main one when developing an FBR [34]. High porosity allows recipient cells to penetrate into deeper layers of the implant and increases the area of interaction with surface chemical groups. Considering that amines, which are present in collagen in large quantities, provoke a more active cellular attack compared to other groups [34], the severity of an FBR in collagen implants can be explained. The weak FBR in REPEREN implants can be explained by the fact that it is a non-porous film, impermeable to recipient cells, which form a very thin fibrous capsule on the surface of the implant by day 30. This capsule isolates the material from further cellular attacks (Figure 5B,D,E). In addition, methacrylate derivatives themselves are quite bioinert [35].

Based on the above, the presence of suture material is the main factor provoking collagen mineralization in the DE-treated pericardium. Although an ideal suture material should not cause a foreign body reaction [36], all currently available suture materials induce it, which subsequently results in calcification. This phenomenon is known for nylon, polydioxanone, polyglactin, polyethylene terephthalate, etc., and the substrate for mineralization can be not only devitalized xenogeneic tissues but also native recipient ones (e.g., arteries, soft tissues, skin) [35,36,37,38]. Most tissue-engineered right-sided valved conduits show calcification along the suture line [39]; the authors consider active reaction toward suture material as one of the main hypothesized mechanisms for calcification.

The key factor determining the severity of an FBR and its outcome is the M1 and M2 macrophages interaction. The classic concept attributes a pro-inflammatory effect to M1 and an anti-inflammatory effect to M2 macrophages [33,34]. However, in recent years this concept has been revised. Indeed, the M1 phenotype produces a large number of pro-inflammatory cytokines, integrins, and other signaling molecules, as well as matrix metalloproteinases (MMPs) [33,34,40]. MMPs are a key for the degradation and lysis of collagen [40], which becomes the main substrate for mineralization. This is facilitated by the acidic inflammatory environment and a large number of different reactive groups released during collagen hydrolysis. At the same time, it has been shown that the development of ectopic calcification can be promoted by the extracellular DNA of M2 macrophages [41]. M1 macrophages are considered responsible for microcalcification and M2 macrophages for macrocalcification [40], although this division is rather nominal.

In relation to our work, it can be assumed that all types of suture material stimulate an FBR in the collagen implant and also, apparently, MMP production. The extensive cellular infiltration of the deep layers of the DE pericardium leads to the coverage of larger areas of collagen undergoing lysis. Degraded collagen serves as a substrate for the nucleation of calcium phosphate. When using PET and PTFE, the FBR fades by the 90th day, cellular infiltration decreases, and the Ca/P ratio in calcium deposits corresponds to that in mature hydroxyapatite (Table 1), which indicates the completion of crystal formation. The most unfavorable results were obtained with PP: the activity of the cellular response, the “spotty” pattern of collagen mineralization (Figure 7E,F), and the mosaic structure of calcium phosphate deposits (Table 1) persist to the 90th day, which indicates the potential for further crystal formation. In addition, these samples accumulated the highest concentration of calcium compared to the cross-linked PET and PTFE samples (Figure 4).

Based on the obtained results, we consider the use of PET to be optimal in the manufacture of pericardial conduits. DE pericardium sutured with PET accumulates the least amount of Ca; the active phase of the FBR practically ends by the 90th day. The use of polypropylene is undesirable, since it, on the contrary, provokes a more pronounced and prolonged FBR and the maximum accumulation of calcium in the implants. As for the film made of the synthetic polymer REPEREN, both PET and PP can be used in its production: both of these materials do not cause calcification in the suture area.

It is not yet possible to formulate a definitive opinion about PTFE sutures. Histological results show no difference in the expression of the FBR in pericardial samples when using PET or PTFE; however, PTFE stimulates the greater accumulation of calcium (Figure 4). At 60 days after implantation, this difference is significant (*p* = 0.002), although by the 90th day it remains only at the level of an insignificant (*p* = 0.296) tendency. A similar picture is observed in the REPEREN samples, although the calcium concentration in them is clinically insignificant [32]; Ca accumulation occurs in the tissues surrounding the implant and in the absence of an active FBR. This may be associated with the possible tissue toxicity of the material itself; at least, the debate on this issue continues, and an increasing number of studies show such toxicity [42,43,44,45]. If we proceed only from the activity of the FBR and the mineralization of the implant, then in the manufacture of cardiovascular prostheses from the non-porous REPEREN film, it is possible to allow the use of all three types of suture material studied.

In this work, we have shown only the “macro” effects of three suture materials. Certainly, to explain the obtained results, a detailed analysis of the cellular components and molecular structures involved in the FBR and the accumulation of calcium during the interaction of the collagen biopolymer/suture polymers is necessary.

## 5. Conclusions

1. The DE-treated bovine pericardium subcutaneously implanted in rats exhibits a severe FBR without calcification. Any suture material in the implant intensifies the FBR, leading to the lysis and homogenization of collagen near the suture, followed by the calcification of these areas.
2. The highest calcium content is found in these collagen implants, sutured with polypropylene. The use of polyester and polytetrafluorethylene allows us to obtain better results.
3. Compared with xenogeneic collagen, the non-porous film made of the synthetic polymer REPEREN shows s very weak FBR and no calcification in both the control and sutured samples.
4. Manufacturing cardiovascular collagenous bioprostheses with polyester suture material can be recommended; prostheses from REPEREN can be sutured with any of the three materials studied.

## Figures and Tables

**Table 1:** Qualitative and quantitative characteristics of calcium deposits in the studied BP samples.

Material Combinations		Localization	Structure (Min and Max Ca/P Ratio)	Ca/P Ratio
Sample Material	Suture Material			
30 days				
DE-treated bovine pericardium	none (control)	-	-	-
	polyester	near the ligature	“mosaic” (from 0.17 to 1.68)	0.93 (0.58; 1.26)
	polypropylene	near the ligature	“mosaic” (from 0.32 to 9.42)	1.30 (1.20; 1.48)
	PTFE	near the ligature	“mosaic” (from 0.29 to 11.41)	1.57 (1.41; 2.73)
60 days				
DE-treated bovine pericardium	none (control)	-	-	-
	polyester	inward deposit growth	“mosaic” (from 0.24 to 13.49)	1.48 (1.30; 2.77)
	polypropylene	inward deposit growth	“mosaic” (from 0.74 to 3.39)	1.37 (1.23; 1.50)
	PTFE	in width and depth of the implant	homogenous	1.70 (1.62; 1.82)
90 days				
DE-treated bovine pericardium	none (control)	-	-	-
	polyester	in deep layers	homogenous	1.69 (1.62; 1.72)
	polypropylene	in deep layers	“mosaic” (from 0.98 to 4.14)	1.40 (1.31; 1.55)
	PTFE	in deep layers	homogenous	1.68 (1.65; 1.69)
REPEREN^®^	none (control)	-	-	-
	polyester	connective tissue capsule	P predominance	0.60 (0.51; 0.67)
	polypropylene	connective tissue capsule	P predominance	0.21 (0.14; 0.35)
	PTFE	connective tissue capsule	P predominance	0.61 (0.49; 0.63)

## Data Availability

The data presented in this study are available on request from the corresponding author, due to the privacy agreement between the authors and the E. Meshalkin National Research Center.

## Supplementary Materials

The following supporting information can be downloaded at https://www.mdpi.com/article/10.3390/polym17111576/s1. Figure S1: Original CT images (A,C) and 3D reconstructions (B,D) demonstrating the suture line calcification in the pericardial right-sided valved conduit. Patients are 19 years old, underwent RVOT reconstruction in tetralogy of Fallot 5 years ago (A,B) and 43 years old, underwent the Ross procedure with a DE-treated pericardial conduit 7 years ago. The valved conduit in the pulmonary artery position is enclosed in a circle. Arrows point to calcium deposits. CT images were obtained from the archives of the E. Meshalkin Medical Research Center. (E) Intact DE-treated pericardial conduit manufactured with polyester sutures (NeoCor company, Kemerovo, Russia). Figure S2: Precision CO_2_ laser cutting machine “MELAS-Cardio” (Institute of Laser Physics of the Siberian Branch of the Russian Academy of Sciences, Novosibirsk, Russia). The red arrow points to a platform for the plate material setting. The yellow arrow points to a contact material thickness measurement sensor; the green one points to a laser radiation source. Figure S3: The 30-day-sutured REPEREN implants. H&E (left column) and von Kossa (right column) staining. Suture materials: polyester (A,B), polypropylene (C,D), and polytetrafluorethylene (E,F). Figure S4: The 60-day-sutured REPEREN implants. H&E (left column) and von Kossa (right column) staining. Suture materials: polyester (A,B), polypropylene (C,D), and polytetrafluorethylene (E,F). Figure S5: The 90-day-sutured REPEREN implants. H&E (left column) and von Kossa (right column) staining. Suture materials: polyester (A,B), polypropylene (C,D), and polytetrafluorethylene (E,F). Figure S6: EDS map of the 90-day REPEREN implant sutured with polyester; spectrum below reflects the Ca/P ratio 0.16.

## Abbreviations

The following abbreviations are used in this manuscript:

BP	bovine pericardium
FBR	foreign body reaction
FBGCs	foreign body giant cells
DE	ethylene glycol diglycidyl ether
PE	polyester
PP	polypropylene
PTFE	polytetrafluorethylene
SEM	scanning electron microscopy
EDS	energy dispersive spectrometry
Ca	calcium
P	phosphorus
