# Supplementary material for: Biopolymer/Suture Polymer Interaction: Is It a Key of Bioprosthetic Calcification?
Source: Polymers (Basel). 2025 Jun 5;17(11):1576. doi: 10.3390/polym17111576 (PMC12157850; doi:10.3390/polym17111576)
Supplement: Supplementary file 1 [file polymers-17-01576-s001.zip › polymers-3530156-supplementary.pdf]

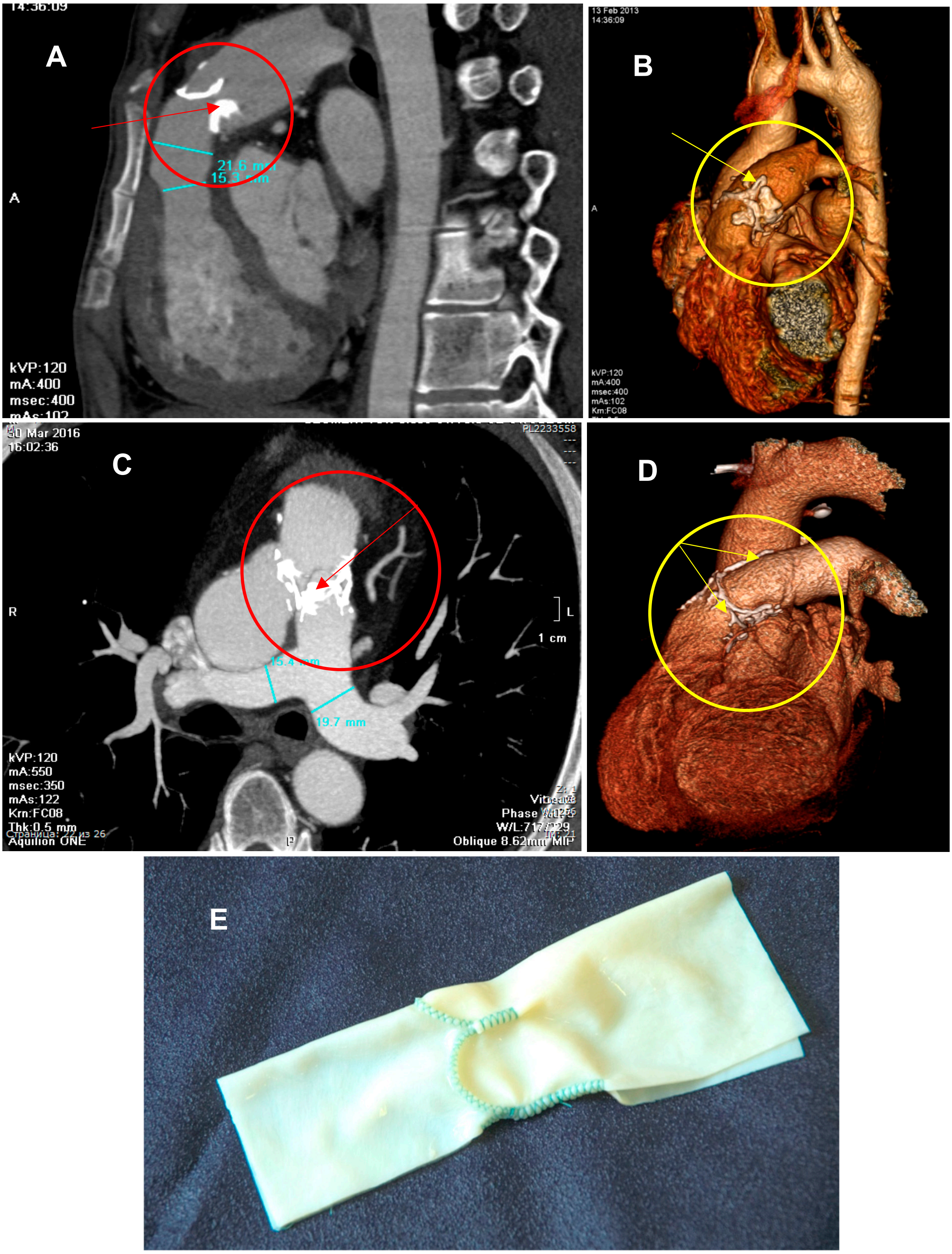

Figure S1. Original CT images (A, C) and 3D reconstructions (B, D) demonstrating suture line calcification in pericardial right-sided valved conduit. Patients are: 19 years old, underwent RVOT reconstruction in tetralogy of Fallot 5 years ago (A, B) and 43 years old, underwent Ross procedure with DE-treated pericardial conduit 7 years ago. Valved conduit in pulmonary artery position is enclosed in a circle. Arrows point to calcium deposits. CT-images were obtained from the archives of the E. Meshalkin Medical Research Center. (E) – intact DE-treated pericardial conduit manufactured with polyester sutures (NeoCor company, Kemerovo, Russia).

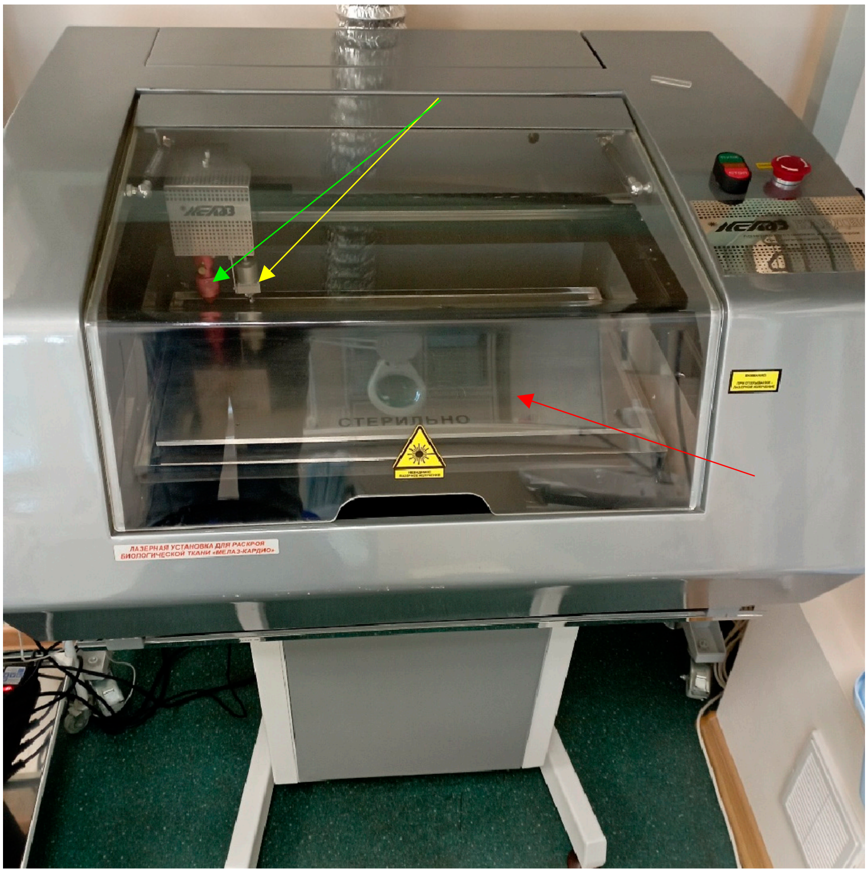

Figure S2. Precision CO_2 laser cutting machine "MELAS-Cardio" (Institute of Laser Physics of the Siberian Branch of the Russian Academy of Sciences, Novosibirsk, Russia). Red arrow points to a platform for plate material setting. Yellow arrow points to a contact material thickness measurement sensor; green one points to a laser radiation source.

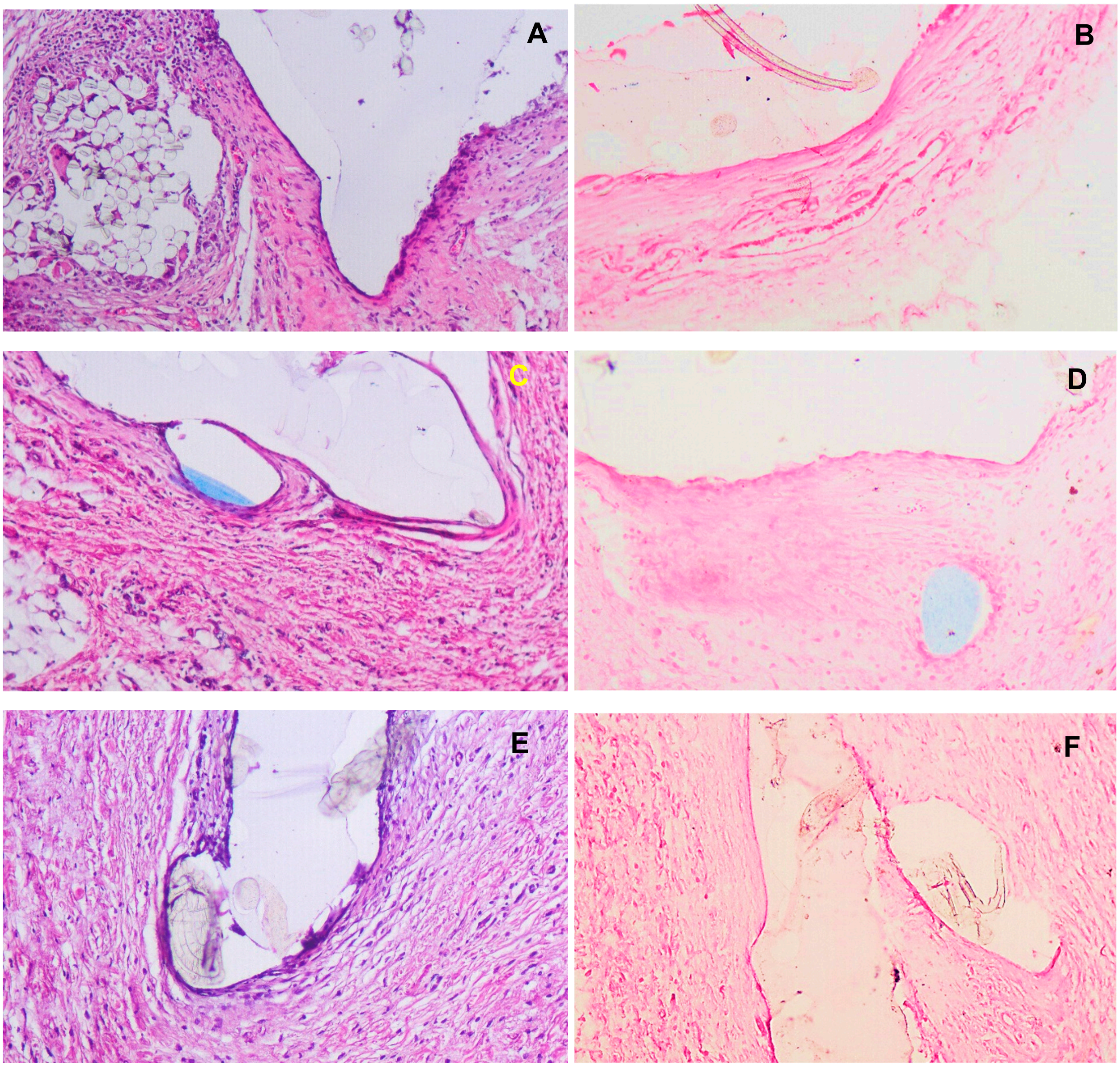

Figure S3. The 30-days sutured REPEREN implants. H&E (left column) and von Kossa (right column) staining. Suture materials: polyester (A, B), polypropylene (C, D), and polytetrafluorethylene (E, F).

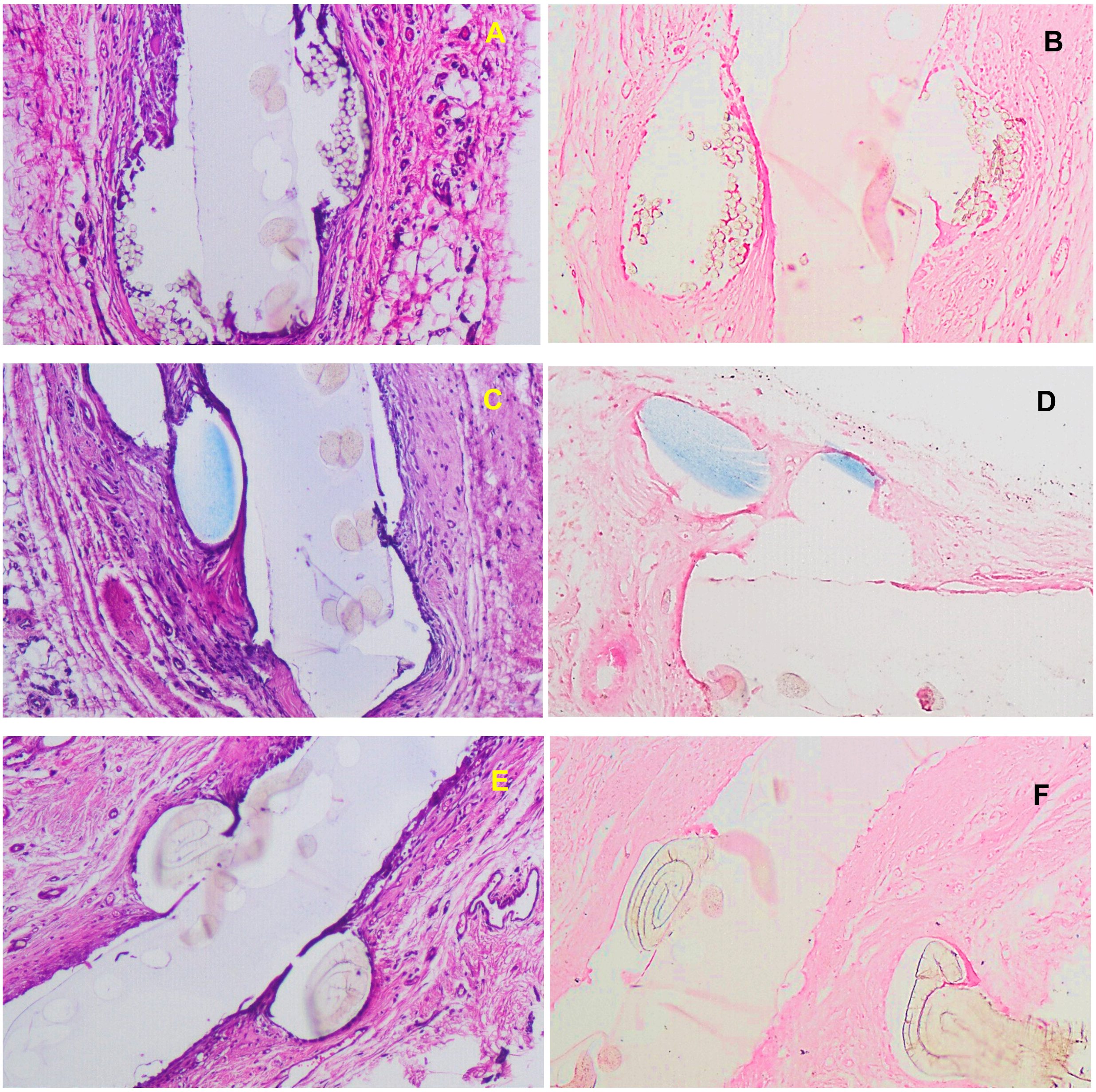

Figure S4. The 60-days sutured REPEREN implants. H&E (left column) and von Kossa (right column) staining. Suture materials: polyester (A, B), polypropylene (C, D), and polytetrafluorethylene (E, F).

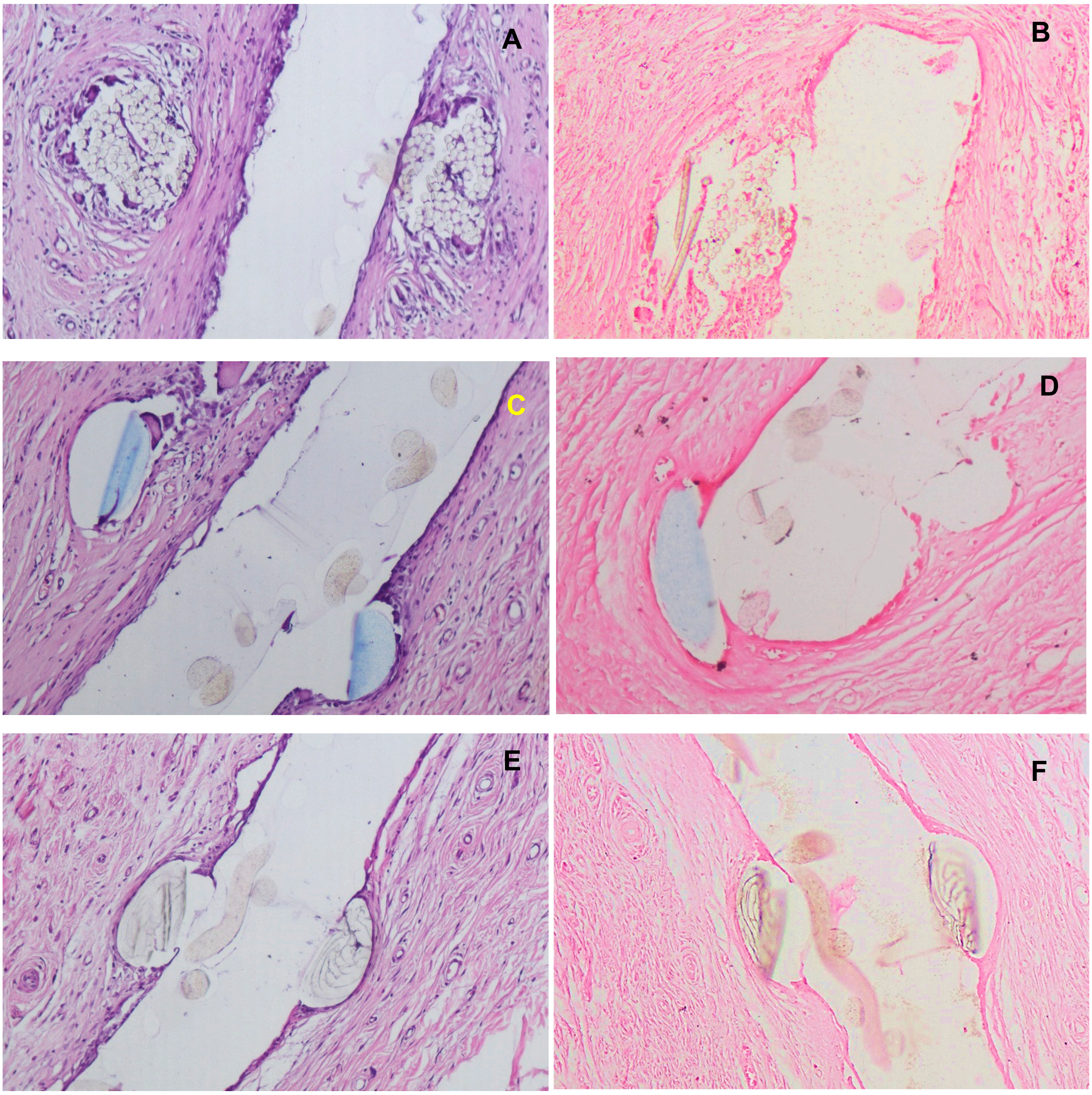

Figure S5. The 90-days sutured REPEREN implants. H&E (left column) and von Kossa (right column) staining. Suture materials: polyester (A, B), polypropylene (C, D), and polytetrafluorethylene (E, F).

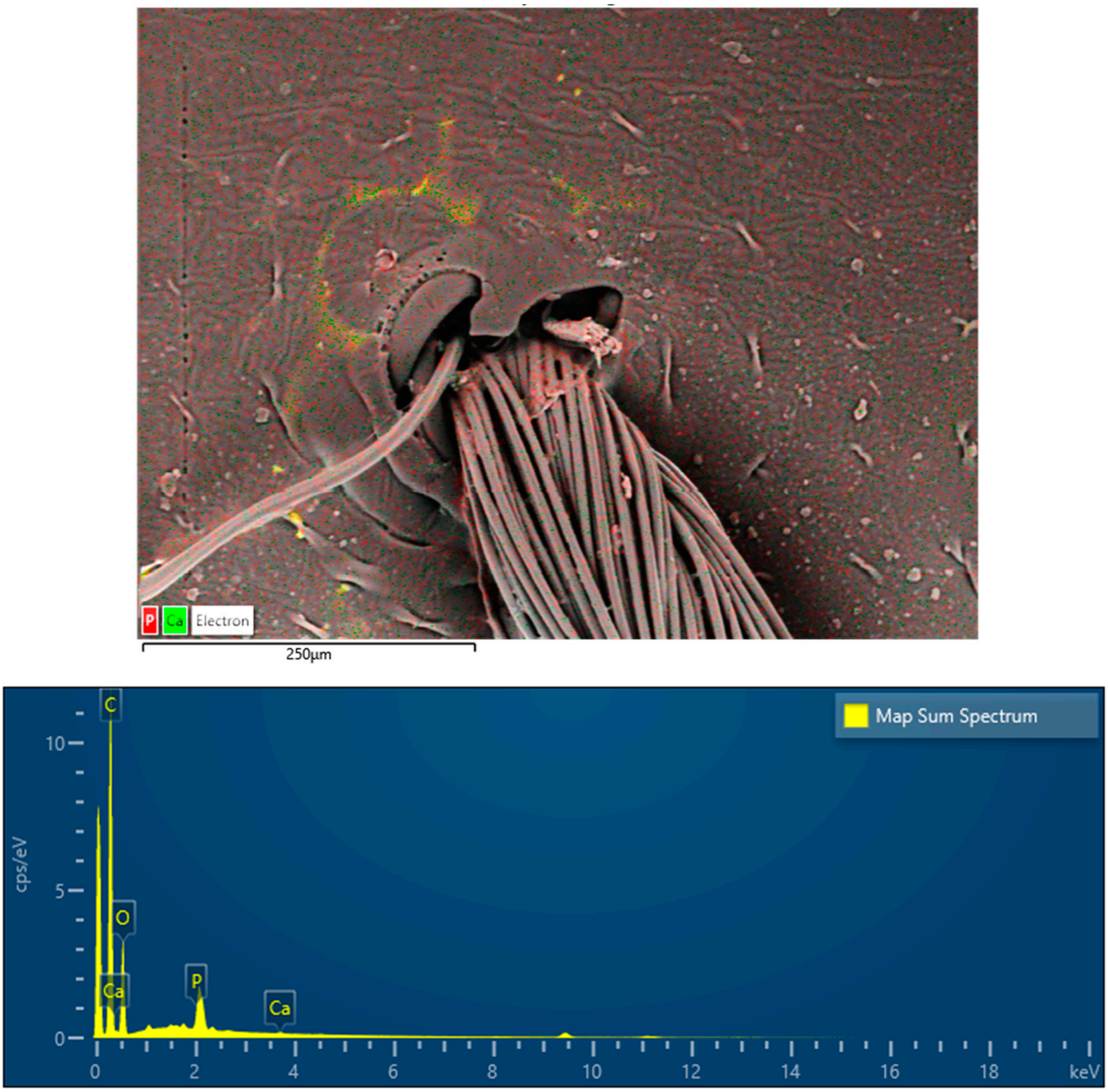

Figure S6. EDS map of the 90 days REPEREN implant sutured with polyester, spectrum below reflects the Ca/P ratio 0.16.
